# Localized granuloma annulare successfully treated with ruxolitinib

**DOI:** 10.1016/j.jdcr.2025.10.057

**Published:** 2025-11-06

**Authors:** Julianna Shin, Marie Leger

**Affiliations:** a Department of Dermatology, Touro University California, Vallejo, California; b Department of Dermatology, Mount Sinai, New York, New York

**Keywords:** granuloma annulare, JAK inhibitor, ruxolitinib, topical therapy

## Introduction

Granuloma annulare (GA) is a cutaneous, granulomatous skin disorder with unknown etiology. While typically asymptomatic, its clinical presentation can cause distress, prompting patients to seek treatment. Traditional therapies have shown modest success, leading to the exploration of new treatment options.

## Case report

**Fig 1:**
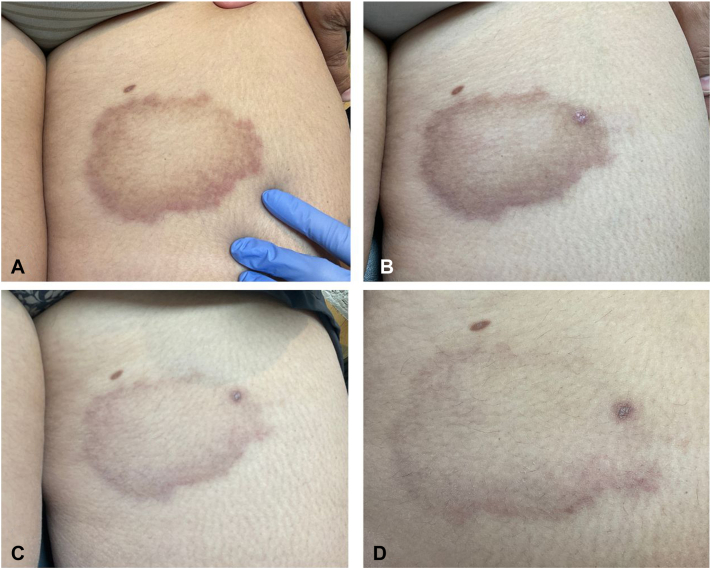
**A,** Initial presentation demonstrating 1 annular patch on the left proximal lower extremity **(B)** presentation at 4-week follow-up **(C)** presentation 6-weeks later **(D)** presentation 12-weeks later.

**Fig 2:**
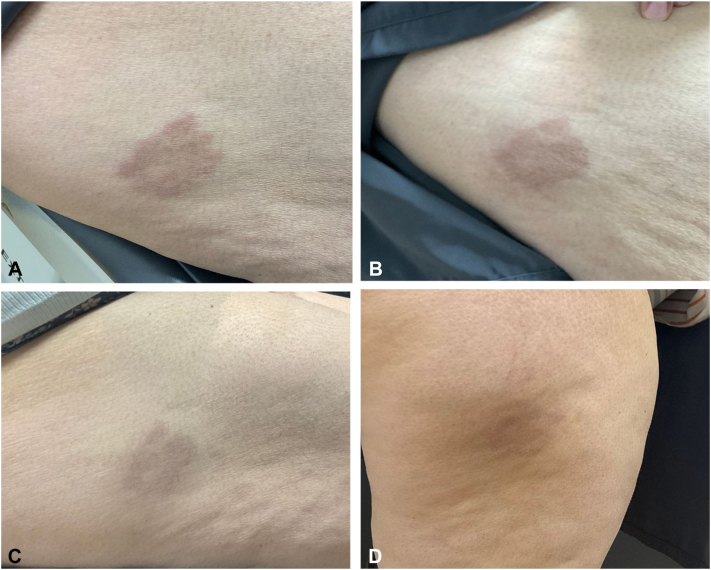
**A,** Initial presentation demonstrating 1 annular patch on the right lateral lower extremity **(B)** presentation at 4-week follow-up **(C)** presentation 6-weeks later **(D)** presentation 12-weeks later.

**Fig 3:**
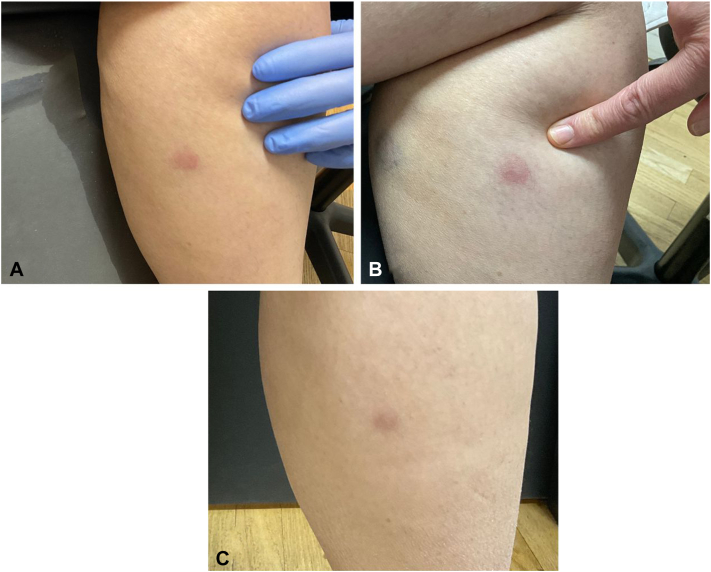
**A,** Initial presentation demonstrating 1 annular patch on the left dorsal lower leg **(B)** presentation at 4-week follow-up **(C)** presentation 12-weeks later.

A 46-year-old female presented to dermatology for the evaluation of a rash involving her left proximal lower extremity, right lateral upper extremity, and the left dorsal lower leg, present for 3 years without any associated symptoms. The patient reported no significant dermatologic history, but reported she had been diagnosed and treated for breast cancer 1 year prior. A physical exam revealed multiple annular hyperpigmented patches on the left proximal lower extremity, the right lateral lower extremity, and the left dorsal lower leg. A punch biopsy was performed on the left proximal lower extremity which showed granuloma annulare with histiocytes and lymphocytes present in the dermis between collagen bundles and surrounding foci of degenerated collagen and increased mucin. The patient was initially prescribed clobetasol 0.05% ointment to treat the rash. At follow-up at 4 weeks, the patient denied any improvement with clobetasol use. The patient was then prescribed topical ruxolitinib 1.5% cream. At follow-up 6 weeks later, the patient reported significant improvement in the pigmentation of rash at all 3 sites with twice daily ruxolitinib use. Further improvement was noted at the 12-week follow-up after continued use of ruxolitinib (Fig 1, Fig 2, Fig 3).

## Discussion

Granuloma annulare has an incidence of 0.04% and affects both children and adults. GA is characterized by erythematous, nonscaly annular papules and patches with granulomatous inflammation. While the cause of GA is unknown, GA has been associated with systemic conditions such as HIV, diabetes, dyslipidemia, malignancies, thyroid disease, and certain drug exposure.^1^ Histologically, GA is marked by dermal mucin deposition and an interstitial granulomatous inflammation pattern.

Localized GA, the most common subtype, typically presents with limited annular patches, often located on the hands and feet, often resolving spontaneously after 2 to 3 years. The first-line therapy for localized GA is topical and intralesional corticosteroids. However, corticosteroid steroid therapy has varied responses and can lead to cutaneous atrophy and hypopigmentation, particularly distressing for skin of color patients. Alternative treatment options include phototherapy and systemic treatment with methotrexate, antimicrobials, antimalarials, or aprelimast.^2^

Emerging research highlights Janus kinase (JAK) inhibitors as a promising treatment option for GA. One study found significant improvement in localized GA lesions after 15-week use of topical tofacitinib 2% ointment twice daily.^3^ Another study found significant improvement in localized GA after a 6-week course of oral abrocitinib at a dosage of 150 mg daily.^4^ Multiple studies have also found significant improvement in generalized GA with a 6 month course of oral tofacitinib at a dosage of 5 mg daily.^5^^,^^6^ A new study found significant improvement of generalized GA after a 12-week treatment with topical ruxolitinib 1.5% cream.^7^

GA pathogenesis involves upregulation of T-helper cytokines (Th1 and Th2), interleukin-4, and JAK3. Inflammatory cytokines, particularly IFN-γ, activate the JAK-STAT pathway, leading to TNF-α, IFN-γ, and chemokine release, which perpetuate granuloma formation and dermal collagen degradation.^8^ Thus, the JAK pathway amplifies the inflammatory response in localized GA by mediating cytokine signaling, promoting immune cell recruitment, and sustaining granuloma formation.

To our knowledge, our case presents the second successful instance of GA successfully treated with topical ruxolitinib. Ruxolitinib regulates the JAK-STAT pathway by inhibiting JAK1 and JAK2. Although commonly used for atopic dermatitis and vitiligo, ruxolitinib demonstrated significant improvement in pigmentation and lesion appearance in this patient with no hypopigmentation or atrophy. While the lesions did not fully clear, the clinical improvement highlights the potential of topical ruxolitinib as a novel treatment for GA. Further research is warranted to establish the safety and efficacy of topical ruxolitinib for this condition.

## Conflicts of interest

None disclosed.
